# A qualitative study of patients’ perspectives on collaboration to support self-management in routine rheumatology consultations

**DOI:** 10.1186/s12891-016-0984-0

**Published:** 2016-03-15

**Authors:** Emma Dures, Sarah Hewlett, Nicholas Ambler, Remona Jenkins, Joyce Clarke, Rachael Gooberman-Hill

**Affiliations:** Faculty of Health and Life Sciences, University of the West of England, Bristol, UK; Pain Management Unit, North Bristol NHS Trust, Bristol, UK; Academic Rheumatology, University Hospitals Bristol NHS Trust, Bristol, UK; School of Clinical Sciences, University of Bristol, Bristol, UK; Academic Rheumatology, Bristol Royal Infirmary, Bristol, BS2 8HW UK

**Keywords:** Patient-clinician collaboration, Communication, Self-management, Health services research, Patient perspective, Qualitative

## Abstract

**Background:**

Self-management of inflammatory arthritis (IA) requires patients to address the impact of symptoms, treatment, and the psychosocial consequences of a long term condition. There are several possible mechanisms for facilitating self-management, including patient-clinician interactions in routine consultations. This requires patients to collaborate in their healthcare, and clinicians to specifically encourage and help patients to do so. To design training that enables clinicians to support patients to be actively involved and self-manage requires understanding both patients’ and clinicians’ perspectives about what is important and feasible. Previous research explored the perspectives of clinicians who had undertaken brief training which they were putting into practice in their routine consultations. This study explored the perspectives of patients attending those routine consultations to identify aspects of the interaction that influenced collaboration and self-management.

**Methods:**

Nineteen patients with IA who had attended a routine consultation with a rheumatology clinician at one of four hospitals in England took part in semi-structured interviews. Interviews were transcribed, anonymised and analysed using inductive thematic analysis.

**Results:**

Three themes encompass participants’ thoughts about interactions that facilitated collaboration in consultations and their ability to self-manage their IA: first, patients and clinicians viewing care as a shared endeavour, including patients responding actively to their IA and clinicians exploring and negotiating with patients; second, the need for clinicians to understand the challenges faced by patients, appreciate the impact of IA and focus on patients’ priorities; and third, clinicians using an open communication style, including the use of non-didactic, patient-centred approaches. A fourth theme was perceived benefits of actively engaging in consultations, including increased confidence to deal with the impact of IA and greater acceptance of a long term condition.

**Conclusions:**

Patients perceive that self-management can be facilitated when clinicians and patients view healthcare as a shared responsibility, underpinned by clinicians as experts in the disease and patients as experts in living with it. Clinicians can support patients’ self-management by using non-didactic communication skills to identify patients’ priorities, and to prompt patients to problem-solve and share in setting the consultation agenda. This should inform skills-training for rheumatology clinicians.

**Electronic supplementary material:**

The online version of this article (doi:10.1186/s12891-016-0984-0) contains supplementary material, which is available to authorized users.

## Background

Inflammatory arthritis (IA) refers to a group of multi-systemic, rheumatic conditions characterised by pain, joint swelling and stiffness, and fatigue [1, 2]. Challenges for patients with IA include unpredictable fluctuations in symptoms, functional disability, reduced participation in valued activities, emotional consequences, and complex medication regimens [3]. Treatment comprises pharmacological, non-pharmacological, and surgical interventions to control symptoms, reduce pain, prevent joint and bone damage, and improve mobility and function [4]. In the UK, treatment is typically provided in secondary care by multi-disciplinary rheumatology teams, including physicians, nurse specialists, occupational therapists, and physiotherapists.

Patients' health outcomes and psychological status can improve when they have the information, skills, and confidence to address the impact of their condition and make lifestyle adjustments, for example activity pacing to help manage pain and fatigue [5, 6]. This is known as self-management, and it presents three tasks for patients: medical management (e.g. interacting with clinicians, adhering to medications); role management (e.g. adapting to changes in relationships and social roles); and emotional management (e.g. processing negative emotions such as anger and guilt) [7]. Self-management approaches are often underpinned by the concept of self-efficacy, an individual’s belief in their ability to feel in control, and their willingness to take on and sustain helpful behaviours [8, 9]. Deciding on how best to implement and continue with self-management is rarely a choice that patients can make alone. One important influence is the dynamic created between patients and clinicians [10].

There is evidence that self-management of rheumatic disease can be cost and clinically effective [11], and access to support is recommended in UK, European, and American rheumatology treatment guidelines [4, 12–15]. Currently, there are two main models of self-management support provision: group-based patient education programmes and clinician-patient collaboration within routine consultations [16–19]. This second model should increase accessibility and sustainability of self-management support, be deliverable where capacity is not available within teams for lengthy group programmes, and benefit patients who would not attend a group. However, available data suggest that provision of self-management support is patchy across long term conditions [20, 21]. Within rheumatology there is evidence that patients want support to self-manage; for example, a UK survey with ≥1200 patients with IA found that 82 % wanted support to manage the impact of pain and fatigue and 57 % to manage emotions, and 66 % reported that they would access a self-management support clinic [22]. However even though clinicians view such provision as part of their remit, they often lack appropriate training and clinical time [23].

An integral element of patients’ self-management is active collaboration in their healthcare. To design training that enables clinicians to support patients to take on an active role requires understanding both patients’ and clinicians’ perspectives about what is important, helpful and feasible [24]. Clinicians’ perspectives have been explored in research with rheumatology physicians, nurses, physiotherapists and occupational therapists who have undertaken a variety of brief training (details of which are provided in a previous publication) [25]. For many, finding appropriate training had been a challenge and they would prefer future training to be rheumatology-specific. However, they believed that their routine consultations were enhanced as a consequence of the skills that they learnt. These included cognitive-behavioural techniques, shared agenda setting, and goal setting. This study explored the perspective of patients who attended routine consultations with these clinicians post-training. The objective was to gain insight into their experiences and obtain their views on collaboration and self-management in the context of the routine consultation.

## Methods

Qualitative methods were used as the study aimed to explore patients’ experiences and views. Data were collected using semi-structured interviews based on an interview schedule (see Additional file 1) designed by the research team, comprising patient partners, rheumatology and psychology clinicians, and qualitative methodologists.

### Ethics, permissions and consent

The study was approved by the NRES Committee North East (reference: 12/NE/0068) and the University of the West of England, Bristol research ethics committee (reference: HLS/12/02/24).

Rheumatology clinicians (two rheumatology consultants, one nurse specialist and one occupational therapist, each based at a different hospital in England) who had undertaken brief training to support patient self-management identified potential participants [25]. The clinicians each handed out approximately 15 invitation packs to patients attending consultations during a week-long period. The packs included a cover letter, information sheet and reply slip. To avoid the risk that patients might agree to take part to please their clinician, the cover letter explained that these clinicians were not directly involved in the study, would not know which patients had taken part, and would not have access to study data. Patients were eligible to take part if they were over 18 years old, had a confirmed diagnosis of IA and had capacity to consent. Patients who were interested in taking part responded directly to the study’s lead researcher, ED.

Participants provided written informed consent before taking part in a face-to-face interview at the hospital site where they had attended their consultation. Interviews were conducted by ED, who had no prior relationship with the participants. There was no-else present during the interviews. Interviews were audio-recorded, transcribed and anonymised. All names in this article are pseudonyms.

An inductive thematic analysis was conducted [26, 27]. In the first stage we coded the data by reading each transcript multiple times and making notes in the margins of words or short phrases that captured what was being said by the participant. In the second stage, we collected all of the words and phrases from all of the transcripts onto a clean set of pages. We reduced and refined the list by removing duplications and merging overlapping codes. We then grouped conceptually-related codes together to inform our overarching themes and sub-themes. This was an iterative process, in which we constantly compared the emergent themes and sub-themes with the transcript codes.

Analysis verification can make the study findings more rigorous and reduce researcher bias [28]. Therefore all transcripts were analysed by the lead researcher and a sub-set of six were analysed independently by three members of the study team. The final analysis was informed by four team members’ interpretations of the data (ED, RJ, JC, SH).

## Results

Nineteen patients (14 women and five men) took part. Their ages ranged from 27 to 75 years and they had been diagnosed for between 0.75 and 40 years (Table 1). Three themes captured patients’ thoughts about interactions that influenced their self-management. These three themes were inter-related and fed into a fourth theme which identified the benefits of active engagement in consultations.

**Table 1:** Patient demographics

Name	Age (years)	Diagnosis^a^	DD^b^	Site^c^
Philip	68	RA	20	A
Nicola	38	RA	5	A
Jane	66	RA	15	A
Eva	60	RA	12	A
Barbara	69	RA	2	A
Patrick	59	PsA	40	A
Felicity	55	RA	16	A
Jean	67	RA	6	B
David	39	RA	8	B
Mark	60	RA	2.5	B
Paul	53	RA	6	B
Fiona	75	RA	5	B
Tracy	67	PsA	1	B
Carly	39	RA	0.75	B
Vanessa	63	RA	26	C
Jayne	44	PsA	2	C
Annie	42	RA	2	C
Susan	63	RA	20	C
Josie	27	RA	1	D

^a^ *RA* rheumatoid arthritis, *PsA* psoriatic arthritis; ^b^ *DD* disease duration in years; ^c^Recruiting clinician:

Site A = consultant rheumatologist

Site B = consultant rheumatologist

Site C = nurse specialist

Site D = occupational therapist

### Theme 1: patients and clinicians viewing care as a shared endeavour

Participants explained that they valued the expertise and support of clinicians, but they felt better able to deal with their arthritis when they were actively involved in their care rather than passive recipients of advice and treatment.

#### Patients taking responsibility for their IA and their care

Participants described how they were open with clinicians about how IA affected them. They saw such disclosures as essential means through which to foster good working relationships with clinicians. For instance, some reported value in keeping a record of symptoms and impact, which then became the basis for discussions in consultations. Participants thought that an active response was fundamental for living well with IA, and expressed a sense of individual responsibility for making behavioural and psychological adjustments.

*“I have a responsibility as it is my own care and in terms of kind of getting stuff he’s not a mind reader, so I have to present stuff as well, so that’s the responsibility of the patient, you have to say what’s going on really” [Felicity]*

*“you’re the one who benefits in the long run if you can really keep a good record of how things have been behaving in the in-between time, and then you might recognise a pattern or something and then it might be easier for them to know what sort of treatment is relevant for you” [Janet]*

In addition to reporting their own health, participants needed to feel informed and knowledgeable to engage in their care. This usually involved a willingness to ask questions and clinicians being prepared to answer them.

*“you’ve just got to be proactive … just not being afraid of asking questions and things because at the end of the day it’s kind of, it’s your health” [Josie]*

*“I’ve certainly always felt that I can ask questions and I can weigh up the options… I don’t feel I’ve been dictated to” [Felicity]*

#### Clinicians exploring and negotiating with patients

This view of care as a shared endeavour did not bring an expectation that the clinicians would always have definitive answers, however participants described the importance of clinicians’ willingness to explore options and to be open to negotiation.

*“although he [consultant] couldn’t give me the full answer, I didn’t go away, as I said earlier from my GP practice thinking, ‘They don’t understand’ or something like that, whereas he understood, and we explored it together” [Eva]*

*“he’s [consultant] open to change an idea if something isn’t working….it’s a fractional difference but, you know, one thing being attentive and listening, another thing taking it on-board and being prepared to change one’s own opinion as a result of that, you know” [Philip]*

As well as openness and collaboration between patients and clinicians, participants thought that it was beneficial when different healthcare professionals worked as a team.

*“I have been referred on, you know, to various people, which perhaps wasn’t the pathway, but it’s what I needed at that time… So I’ve always felt that, that I could tell them anything and then they would send me off to who I needed to see if they didn’t feel they could manage it at that point… but they are acknowledging that it’s something that’s an issue to you and trying to help you find somebody that can help you with that” [Annie]*

### Theme 2: clinicians understanding the challenges faced by patients

As part of viewing care as a shared endeavour, participants valued clinicians understanding the ways in IA affected them.

#### Appreciating the impact of the condition

It was important to participants that clinicians did not focus solely on the symptoms of IA and their pharmacological treatment. Participants thought that clinicians needed to acknowledge and seek to understand the range of physical, social and emotional consequences that participants were dealing with. This holistic focus contributed to the perception that clinicians were caring and attentive.

*“The impact on work, on my career, on my finances, you know, on times that my children have had to become my carers, you know, there’s more to just having arthritis than just taking drugs… look at the bigger picture, to not just look at the diagnosis, to look at the long-term effects that this condition has on somebody’s life” [Jayne]*

*“I can tell her sort of like things like, she’ll say to me, “What do you find distressing about it?” I mean I’ve got a recently new grandson, and I can’t pick him up, and that upsets me ‘cos I – I can’t hold him”[Tracy]*

#### Focusing on patients’ priorities

Participants thought that discussions in consultations enabled clinicians to understand and address their main concerns, questions and priorities.

*“it was very much focused on my needs and what I felt I wanted out of the consultation, my specific work issues and sort of backed up with a general chat about pacing and drug management so it was very much led by my concerns” [Annie]*

### Theme 3: open, non-didactic communication

Participants appreciated how open, patient-centred communication styles, rather than didactic approaches, helped to create the sense of shared responsibility.

#### The use of non-didactic, patient-centred approaches

Consultations were constructive when clinicians prompted participants to think through a challenge, identify a priority for change and set goals, or try and come to their own decision, rather than telling them what to do.

*“it was just suggesting different ways, but without telling me almost, it was making me think for myself how best I can do it. And I suppose that would be going towards thinking, not just for that particular task that I couldn’t do, it’d make me think about something else that I was doing as well” [Vanessa]*

*“no-one pushes you into anything, or anything like that, you know. It’s a case of, ‘How do you feel? How do you think? Could you benefit from that?’… Yeah they can talk around it and then you make the decision” [Fiona]*

*“talking about goals, and goal-setting that was helpful, we talked a bit about what I was wanting, you know, what was my main concern and how I might do things differently and what I would need to do for that to work for me, and that was good ‘cos it got me thinking” [Josie]*

Along with the two-way dialogue, the incorporation of visual materials and written records into the consultation aided communication and understanding.

*“I can give any information, she’s giving me feedback, I’m maybe giving counter-feedback, everything’s written up, we agree on the way forward” [Paul]*

*“he [consultant] uses a piece of paper and pencil, so you get a visual information whilst he’s talking, so, and that I think is helpful as well” [Felicity]*

### Theme 4: the benefits of active engagement in consultations

Becoming a patient with IA and finding ways to self-manage was challenging. Participants felt that routine consultations were an important source of support when they addressed the impact of IA from their perspective and when collaboration focused on optimising care and well-being. Participants identified benefits of actively engaging in consultations.

#### Increased confidence to deal with the impact of IA

When participants interacted with clinicians who encouraged patient activation and viewed care as a collaborative endeavour, it enabled them to challenge previously held assumptions about the dynamics between professionals and patients. This could increase participants’ confidence to be proactive in the consultation and to value their own experience and expertise, which enhanced their ability to deal with the impact of IA.

*“I feel confident now to ask the questions, and that is, I think, partly to do with the fact that I’ve been put at my ease within consultations” [Vanessa]*

*“it was a question of developing a way of what works best for me, and what I can get out of it when I go into the consultant room. Because before I might have been quite passive and just listened… and think the consultant is kind of up on the pedestal. I still respect him and, you know, he has all the knowledge on that side of things, but I have the knowledge of my body, I’ve realised that, I’ve worked it out” [Eva]*

*“in some ways my life has changed massively already, in that I’m a lot more flexible about how I can deal with this, my health” [Annie]*

#### Greater acceptance of a long term condition

Self-management was described as an on-going process which required thought and effort to sustain. For many participants there had been an important shift from struggling with symptoms, worrying about the future, or a wishing for a cure, to an acceptance of the condition and the belief that it would be manageable.

*“you need to think laterally sometimes, you know, and so I mean at the moment my mornings are quite difficult, so I’m thinking, ‘OK I’ll leave that till this afternoon when I know things will improve’… and I’m still struggling with that, even though I’m telling you everything I’ve learnt but I’m getting there” [Vanessa]*

*“I mean it still hurts, it’ll never ever go and there’s lots of things now that I can’t do, but I’ve accepted it now where I wouldn’t accept it before” [Susan]*

*“I think it was a lot about ‘What’s my future going to be like? Is there a cure? Are the kids gonna have it? This is what I’m gonna be like.’ Now I think the focus is much more on sort of the lifestyle, adapting, OK this is what I’m like now, you know, I need to adapt” [Carla]*

## Discussion

These findings support models of healthcare in which patients are active in the clinical consultation [29–31]. Closely related is the idea of self-management as a process that can be facilitated through collaboration with clinicians [32]. This is underpinned by the recognition of clinicians as experts in the condition and patients as experts in living with it. Both perspectives are important to optimise health outcomes at the individual level and in the delivery of sustainable healthcare systems [33]. Although it has been argued that some patients do not wish to, or cannot, engage in self-management [34], this study found that patients with IA supported the idea of collaboration. However, several participants described how gaining the confidence to be proactive rather than passive took time.

A systematic review confirmed that knowledge alone (e.g. of treatment and personal preferences) is insufficient for patients to feel able to participate in their healthcare, and that patients often undervalue their experiential expertise relative to clinicians [35]. This is exacerbated by a widely held assumption that “doctor knows best” and suggests an important role for clinicians to communicate in a way that can redress perceived imbalances and aid collaboration. It is further supported by evidence that features of clinician–patient interaction in the consultation, such as exchange of information and shared decision making, can predict health outcomes; including adherence to treatment and mental health [36]. Seven pathways have been proposed as mechanisms through which communication can lead to better health: increased access to care, greater patient knowledge and shared understanding, higher quality medical decisions, enhanced therapeutic alliances, increased social support, patient agency and empowerment, and better management of emotions [37, 38]. The experiences of participants in the current study provide insights into several of these pathways, and provides further evidence that communication is a core component of quality of care [39, 40].

Specifically, participants identified non-didactic approaches as constructive for facilitating engagement, describing how they enabled them to ask questions, problem-solve, and be involved in decision-making. This was fostered by encouragement from clinicians, and fits with the theory of building self-efficacy through social persuasion [5, 8]. In addition, it supports the conceptual model of collaborative deliberation. The model’s authors propose five foundations to facilitate clinical communication processes and patient-centered interactions: constructive engagement; recognition of alternative actions; comparative learning; preference construction and elicitation; and reference integration of individual preferences [41]. Our current study suggests that collaborative deliberation is a helpful theoretical framework which could be underpinned by ccommunication approaches such as motivational interviewing [42], Five Step Patient-Centred Interviewing [43] and Shared decision-making [44, 45].

Participants in this study had attended a consultation with a clinician who had undertaken brief training to support patients’ self-management [25]. In this previous research, these clinicians identified the importance of embedding such support in routine care. One example would be through familiarising members of the rheumatology team with the value and principles of self-management. Involvement of the multi-disciplinary team in supporting patient self-management would not require all clinicians to be equally skilled as they could sign-post patients to colleagues and to appropriate resources outside the team or rheumatology service. These clinicians who had undertaken brief training to support patients’ self-management [25], also identified approaches that seemed effective for increasing patient self-efficacy, for example eliciting patients’ IA-related priorities in the consultation and then addressing their agenda. Findings from this current study with patients support the findings from the study with clinicians. Together they identify benefits of moving from a traditional clinician-centred approach to care to a more holistic, collaborative one.

### Limitations

This was a small-scale study, but the sample of 19 patients was sufficient to achieve data saturation such that no new themes were emerging by the time that data collection was complete. The sample comprised men and women with a range of ages and disease duration. However, all participants were selected on the basis that their treating clinicians had received training in provision of support for self-management, and this means that the patients had experiences that might not reflect those of patients in general. It is also possible that patients who agreed to be interviewed were those with the most positive experience of interactions with their clinicians. To reduce the chance of this, clinicians were asked to invite consecutive patients to take part in the study, but requirements of the ethics approval meant that invitation letters were signed by patients’ clinicians and we are aware that there was some inadvertent selection when clinicians found that information packs were not readily available or if they had other pressing commitments.

## Conclusion

This qualitative study has provided insights into patients’ perceptions about how interactions in routine consultations influenced their confidence to collaborate in their healthcare, a key condition for facilitating self-management. Participants thought it important that patients and clinicians shared responsibility for healthcare and worked together to optimise well-being; that clinicians understood and explored how patients were affected by their IA; and that clinicians communicated in an attentive and non-didactic style. When supported in these ways, participants were more accepting of their IA and confident about self-managing.

The approaches described in this study require both clinicians and patients to work within a model of healthcare premised on shared responsibility and expertise. There is growing evidence of clinicians’ training needs in relation to providing support for self-management and the need for greater understanding of how to help patients take on a more active role. Future research should also develop strategies for embedding self-management support into routine care, including processes to facilitate multi-disciplinary team-wide support.

## Additional file

Additional file 1: Semi-structured interview schedule. (DOCX 12 kb)

## Abbreviations

IA: Inflammatory arthritis

## References

[1] Conaghan PG, Green MJ, Emery P (1999). Established rheumatoid arthritis. Baillieres Best Pract Res Clin Rheumatol.

[2] Young A, Dixey J, Cox N, Davies P, Devlin J, Emery P (2000). How does functional disability in early rheumatoid arthritis (RA) affect patients and their lives?. Rheumatology (Oxford).

[3] Homer D (2005). Addressing psychological and social issues of rheumatoid arthritis within the consultation: a case report. Musculoskeletal Care.

[4] Luqmani R, Hennell S, Estrach C, Birrell F, Bosworth A, Devenport G, et al. British Society for Rheumatology and British Health Professionals in Rheumatology guideline for the management of rheumatoid arthritis (the first 2 years). Rheumatology. 2006;1–16. 10.1093/rheumatology/kel215a 16844700

[5] De Silva D (2011). Evidence: helping people help themselves.

[6] Barlow J, Wright C, Sheasby J, Turner A, Hainsworth J (2002). Self-management approaches for people with chronic conditions: a review. Patient Educ Couns.

[7] Lorig K, Holman H (2003). Self-management education: history, definition, outcomes, and mechanisms. Ann Behav Med.

[8] Bandura A (2001). Social cognitive theory: An agentic perspective. Annu Rev Psychol.

[9] Evers A, Zautra A, Thieme K (2011). Stress and resilience in rheumatic diseases: a review and glimpse into the future. Nat Rev Rheumatol.

[10] Fischer M, Ereaut G (2012). When doctors and patients talk: making sense of the consultation.

[11] Dures E, Hewlett S (2012). Cognitive-behavioural approaches to self-management in rheumatic disease. Nat Rev Rheumatol.

[12] National Institute for Health and Care Excellence. Rheumatoid arthritis: the management of rheumatoid arthritis in adults. CG79 issued February 2009, modified August 2013 (http://www.nice.org.uk/CG79)

[13] Forestier R, Andre-Vert J, Guillez P, Coudeyre E, Lefevre-Colau M, Combe B (2009). Non-drug treatment (excluding surgery) in rheumatoid arthritis: clinical practice guidelines. Joint Bone Spine.

[14] Luqmani R, Hennell S, Estrach C, Basher D, Birrell F, Bosworth A (2009). British Society for Rheumatology and British Health Professionals in rheumatology guideline for the management of rheumatoid arthritis (after the first 2 years). Rheumatology.

[15] American College of Rheumatology Subcommittee on Rheumatoid Arthritis Guidelines (2002). Guidelines for the management of Rheumatoid Arthritis: Update. Arthritis Rheum.

[16] Thoesen M, Newton K (2005). Supporting self-management in patients with chronic illness. Am Fam Physician.

[17] Kennedy A, Rogers A, Gately C (2005). Assessing the introduction of the expert patients programme into the NHS: a realistic evaluation of recruitment to a national lay-led self-care initiative. Primary Health Care Res Dev.

[18] De Silva D (2012). Helping people share decision-making.

[19] Cramm J, Nieboer A (2014). A longitudinal study to identify the influence of quality of chronic care delivery on productive interactions between patients and (teams of) healthcare professionals within disease management programmes. Brit Med J Open.

[20] Newbronner L, Chamberlain R, Borthwick R, Baxter M (2013). Sanderson D.

[21] Coulter A, Roberts S, Dixon A. Delivering better services for people with long term conditions. The King’s Fund. October 2013 (http://www.kingsfund.org.uk/sites/files/kf/field/field_publication_file/delivering-better-services-for-people-with-long-term-conditions.pdf).

[22] Dures E, Almeida C, Caesley J, Peterson A, Ambler N, Morris M (2014). Psychological support provision for adults with inflammatory arthritis in secondary care. Musculoskeletal Care.

[23] Dures E, Almeida C, Caesley J, Peterson A, Ambler N, Morris M, et al. Patient preferences for psychological support in inflammatory arthritis: a multi-centre survey. Ann Rheum Dis ADR Online First, September. 2014;26. 10.1136/annrheumdis-2014-205636 25261572

[24] Ong B, Rogers A, Kennedy A, Bower P, Sanders T, Morden A (2014). Behaviour change and social blinkers? The role of sociology in trials of self-management behaviour in chronic conditions. Sociol Health Ill.

[25] Dures E, Hewlett S, Clarke J, Jenkins R, Ambler N, Gooberman-Hill R (2014). Rheumatology clinicians’ experiences of brief training and implementation of skills to support patient self-management. BMC Musculoskel Dis.

[26] Braun V, Clarke V (2006). Using thematic analysis in psychology. Quali Res Psychol.

[27] Thorne S, Paterson B (2000). Two decades of insider research: what we know and don’t know about chronic illness experience. Annu Rev Nurs Res.

[28] Burnard P, Gill P, Stewart K, Treasure E, Chadwick B (2008). Analysing and presenting qualitative data. Brit Dent J.

[29] Engel G (1977). The need of a new medical model: a challenge for biomedicine. Science.

[30] Von Korff M, Gruman J, Schaefer J, Curry S, Wagner E (1997). Collaborative management of chronic illness. Ann Intern Med.

[31] Carrio F, Suchman A, Epstein R (2004). The biopsychosocial model 25 Years later: principles, practice, and scientific inquiry. Ann Fam Med.

[32] Eaton S, Collins A, Coulter A, Elwin G, Grazin N, Roberts S (2012). Putting patients first: NICE guidance on the patient experience is a welcome small step on a long journey. Brit Med J.

[33] All Party Parliamentary Group (APPG) Report. Patient empowerment: for better quality, more sustainable health services globally. May 2014. http://www.appg-globalhealth.org.uk/

[34] Lagare F, Thompson-Leduc P (2014). Twelve myths about shared decision making. Patient Educ Couns.

[35] Joseph-Williams N, Elwyn G, Edwards A (2014). Knowledge is not power for patients: a systematic review and thematic synthesis of patient-reported barriers and facilitators to shared decision making. Patient Educ Couns.

[36] Fong Ha J, Longnecker N (2010). Doctor-Patient Communication: a review. Ochsner J.

[37] Tongue J, Epps H, Forese L (2005). Communication skills for patient-centered care: research-based, easily learned techniques for medical interviews that benefit orthopaedic surgeons and their patients. J Bone Joint Surg Am..

[38] Street R, Makoul G, Arora N, Epstein R (2009). How does communication heal? Pathways linking clinician–patient communication to health outcomes. Patient Educ Couns.

[39] Murray Cramm J, Nieboer A (2014). A longitudinal study to identify the influence of quality of chronic care delivery on productive interactions between patients and (teams of) healthcare professionals within disease management programmes. Brit Med J Open.

[40] Taylor S, Pinnock H, Epiphanou E, Pearce G, Parke H, Schwappach A, et al. A rapid synthesis of the evidence on interventions supporting self-management for people with long-term conditions: PRISMS - Practical systematic Review of Self-Management Support for long-term conditions. Health Serv Deliv Res. 2014;2(53). ISSN: 20150-4349. 25642548

[41] Elwyn G, Lloyd A, May C, Van der Weijden T, Stiggelbout A, Edwards A (2014). Collaborative deliberation: a model for patient care. Patient Educ Couns.

[42] Rollnick S, Butler C, Kinnersley P, Gregory J, Mash B (2010). Motivational interviewing. Brit Med J.

[43] Fortin A, Dwamena F, Frankel R, Smith R (2012). Smith’s patient-centered interviewing: an evidence-based method.

[44] Stiggelbout A, Van der Weijden T, De Wit M, Frosch D, Légaré F, Montori V (2012). Shared decision making: really putting patients at the centre of healthcare. Brit Med J.

[45] Elwyn G, Frosch D, Thomson R, Joseph-Williams N, Lloyd A, Kinnersley P (2012). Shared Decision Making: A Model for Clinical Practice. J Gen Intern Med.

