# Supplementary material for: A qualitative study of patients’ perspectives on collaboration to support self-management in routine rheumatology consultations
Source: BMC Musculoskelet Disord. 2016 Mar 15;17:129. doi: 10.1186/s12891-016-0984-0 (PMC4793532; doi:10.1186/s12891-016-0984-0)
Supplement: Additional file 1: — Semi-structured interview schedule. (DOCX 12 kb) [file 12891_2016_984_MOESM1_ESM.docx]

**Additional file 1: semi-structured interview schedule**

**Part A: Background information**

1. How long have you been seeing the rheumatology team here?
2. How long have you been seeing this rheumatology professional?

**Part B: Experiences of interaction and communication in the consultation**

1. How would you describe the clinician’s approach and style of communication?
   1. Did you feel it was collaborative (i.e. you went through things together)?
   2. Did you have any help to work out what you might do in relation to your arthritis?
   3. Did you discuss the things you wanted to?
2. How did you respond to the clinician’s approach and style of communicating?
   1. Was it uncomfortable in any way?
   2. Did it make you re-think your attitude to anything?
3. Does their style of consultation seem different to other medical professionals that you see about your arthritis?
4. Can you tell me about any ways in which you find your visits here are helpful?
   1. Is there anything that has helped you in managing, can you give me an example?
5. Can you tell me about any ways in which you find your visits here are not helpful?
   1. Can you give an example of why not?
6. Did your visit here affect how you manage/deal with your arthritis in your everyday life?
7. Based on your experience, what would you say to:
   1. A new patient, who asks you how to get the most out of a consultation?
   2. A new clinician, who asks you how they can be most helpful to patients?

**Part C: Close**

- Are there any issues that we have not talked about that you would like to raise?
- Thank you very much for your time and valuable contribution to the study.
